# Independent Factors of Changes of Ankle-Brachial Index in Peripheral Arterial Occlusive Disease in Elderly Patients with or without Diabetes

**DOI:** 10.3390/ijerph13111103

**Published:** 2016-11-08

**Authors:** Ewelina Bąk, Czesław Marcisz, Monika Kadłubowska, Anna Michalik, Bożena Krawczyk, Dorota Dobrzyń-Matusiak, Sylwia Krzemińska, Tomasz Fiałkowski, Elżbieta Glądys, Agnieszka Drosdzol-Cop

**Affiliations:** 1 Faculty of Health Sciences, University of Bielsko-Biala, 43-309 Bielsko-Biala, Poland; ewelina.bak76@wp.pl (E.B.); mkadlubowska@ath.bielsko.pl (M.K.); amichalik@ath.bielsko.pl (A.M.); bkrawczyk@ath.bielsko.pl (B.K.); 2 Department of Gerontology and Geriatric Nursing, School of Health Sciences, Medical University of Silesia, 40-752 Katowice, Poland; klinwewtychy@poczta.onet.pl; 3 Department of Nursing Propaedeutics, School of Health Sciences, Medical University of Silesia, 40-752 Katowice, Poland; dorotadobrzyn@op.pl; 4 Department of Clinical Nursing Faculty of Health Sciences, Medical University, 50-367 Wroclaw, Poland; s.krzeminska@wp.pl; 5 Department of Rehabilitation, Medical University, Provincial Hospital in Bielsko-Biala, 43-309 Bielsko-Biala, Poland; tomasz_f@poczta.onet.pl; 6 Specialist Psychiatric Health Care Center, 43-309 Bielsko-Biala, Poland; ela.gladys@vp.pl; 7 School of Health Sciences in Katowice, Medical University of Silesia, Chair of Woman’s Health, 40-752 Katowice, Poland

**Keywords:** ankle-brachial index, peripheral arterial disease, diabetes, elderly

## Abstract

Peripheral arterial disease (PAD) belongs to the commonly-occurring pathologies associated with elderly age. A simple tool for defining the severity of PAD is the ankle-brachial index (ABI). The purpose of this research was to determine independent factors of changes of ABI in elderly patients with occlusive PAD disease (PAOD) with and without diabetes. The research was carried out on 49 elderly patients with PAOD, including 29 patients with type 2 diabetes, and 20 patients without diabetes. The concentration of interleukin-6 (IL-6), E-selectin, fibrinogen, and C-reactive protein (CRP) in the blood serum was marked. In all patients, the independent factors of changes of ABI were determined with the use of the multiple logistic regression analysis. Our results show that in the group of patients with PAOD suffering from diabetes, it was demonstrated that the ABI was related to age, the duration of the symptoms of PAD, body mass index (BMI), low-density lipoprotein cholesterol, fibrinogen, and sex (determination coefficient R^2^ = 0.699). In patients with PAOD without diabetes, the ABI was related to age, the duration of the symptoms of PAD, the levels of CRP, E-selectin, high-density lipoprotein cholesterol, and the glomerular filtration rate(determination coefficient R^2^ = 0.844). We conclude that in elderly patients with PAOD with and without diabetes, the participation of independent factors related to the ABI is diversified; in patients with diabetes, the concentration of IL-6 and fibrinogen is lower, and the concentration of E-selectin is higher than in patients without diabetes.

## 1. Introduction

The commonly-occurring pathological conditions of the cardiovascular system include peripheral arterial disease (PAD), which is generally associated with the arteriosclerosis of these vessels. The incidence of this disease increases with age; it has been found that over 10% of the elderly population (60+ years) of the USA is suffering from PAD [1]. The main risk factors for the development of PAD are: Hyperlipidemia, arterial hypertension, diabetes, smoking, and advanced age [1,2]. Metabolic, inflammatory, haemostatic, and rheological factors are involved in the mechanism of this disease [3]. The element which is particularly emphasized in the development of PAD is the participation of the inflammatory reaction biomarkers, whose concentration is increased or shows a tendency to increase in the circulating blood of patients suffering from this disease [4,5,6,7,8]. The metabolic diseases leading to the development of arteriosclerosis—including the arteriosclerosis of the lower extremities—include diabetes [4]. Diabetes (especially type 2 diabetes) is a well recognized risk factor for cardiovascular diseases, and in combination with the occurrence of vascular wall stiffness, it leads to the development of PAD [9]. It is suggested that in the pathogenesis of PAD in patients suffering from diabetes, a significant role is played by pro-inflammatory cytokines and acute-phase proteins; the mentioned elements include interleukin-6 (IL-6), tumor necrosis factor (TNF)-alpha, C-reactive protein (CRP), and fibrinogen [4,6,10,11]. However, Danielsson et al. [12] reported that the development of PAD in patients suffering from diabetes was not associated with the polymorphism of the gene for IL-6, and that the concentration of inflammatory markers was normal in the lower clinical stage of this disease. Only in patients suffering from diabetes with critical ischemia of the lower extremities, the concentration of IL-6 proved to be higher than in such patients without diabetes [12]. In patients with PAD, in the course of diabetes, there was also increased concentration of E-selectin [13,14], which argues for increased endothelial function leading to diabetic angiopathy. It has been shown that the damage to and dysfunction of the endothelium were consequences of postprandial hyperglycemia [15].

A simple and non-invasive tool for determining the severity of PAD is the measurement of the ankle-brachial index (ABI). Compared with an assessment of the pulse or a medical history, the ABI has been found to be more accurate. It has been validated against an angiographically-confirmed disease and found to be 95% sensitive and almost 100% specific [16]. However, the ABI may underestimate the severity of PAD in patients, especially in diabetics with non-compressible vessels due to arterial calcification [17].

The mechanisms of the emergence and development of PAD in patients with and without diabetes are surely differentiated and determined by many independent factors. The need to become familiar with these factors—which may prove to be useful, especially in clinical practice—justifies the undertaking of research concerning the covariance (correlation) between the values of ABI and the parameters determining the development of PAD in patients in advanced stages of this disease. The purpose of this research was to determine the independent factors that result in changes in ABI in elderly patients with and without type 2 diabetes, suffering from peripheral arterial occlusive disease (PAOD) of the lower extremities, taking into consideration the serum concentrations of Il-6 and E-selectin.

## 2. Materials and Methods

### 2.1. Study Subjects

The research was conducted on 49 patients suffering from PAOD of the lower extremities; in the study, there were 29 patients with type 2 diabetes, aged 60–78 years (mean 70.1), including 11 women and 18 men (Group I); and 20 patients without diabetes, aged 60–75 years (mean 65.3), including six women and 14 men (Group II). Patients were selected for the study from among those consecutively attending the clinic who met the inclusion and exclusion criteria described below. The patients included in group I were selected from among 170 randomly-chosen patients suffering from type 2 diabetes aged over 60 years. The patients included in group II were selected from among 130 randomly chosen patients suffering from PAOD of the lower extremities aged 60 years or over. The inclusion criteria for the study were the occurrence of clinical symptoms of ischemia of the lower extremities (which in patients with diabetes lasted for 1–10 (mean 3.9) years, and in patients without diabetes lasted for 1–7 (mean 3.7) years), the values of the ABI ≤ 0.9, the controlled arterial blood pressure (systolic pressure <140 mm Hg, diastolic pressure <90 mm Hg), cholesterol concentration <200 mg/dL, and in patients suffering from diabetes, additionally: pharmacological treatment lasting for at least 12 months and controlled glycemia (fasting plasma glucose <120 mg/dL, glycated hemoglobin (HbA1c) <7.5%). Patients suffering from rest pain and/or the ulceration of the lower extremities were excluded from the study. All the patients included in the research took statins; 28 patients from group I and 19 patients from group II took blood pressure-lowering medications and aspirin. The treatment of diabetes was based on maintaining a proper diet and taking the derivatives of biguanide, sulfonylureas, and insulin. All the people included in the study were patients of the Diabetologic Clinic and Vascular Clinic of the District Specialist Clinic in Wadowice in the years 2014–2015. The control group included 19 healthy volunteers (nine women, 10 men) aged 60–75 years (mean 64.9).

The study excluded patients who suffered inflammation in the past three months, patients taking immunosuppressive medications, glucocorticoids, anti-inflammatory medications, medications affecting the endothelium, and patients diagnosed with cancer, endocrine gland diseases, alcoholism, and those who did not agree to participate in the research. Patients with incompressible arteries were excluded from the study. All the examined patients provided informed written consent for participation in the study. The study was approved by the Bioethics Committee of the Beskidzka Chamber of Physicians in Bielsko-Biala, Poland (Ethical Code 112/11/2013).

### 2.2. Methods

After being included in the study, the following parameters were determined for all patients: Blood serum concentration of IL-6, E-selectin, fibrinogen, CRP, total cholesterol, low-density lipoprotein-cholesterol (LDL-C), high-density lipoprotein-cholesterol (HDL-C), triglycerides, uric acid, creatinine, and the plasma glucose concentration. The blood for the laboratory tests was obtained from one blood collection, from the antecubital vein under fasting conditions between 07:00 a.m. and 08:30 a.m. The blood samples were allowed to clot, and then they were centrifuged at 3500 rpm at room temperature for 10 min (Horizontal Centrifuges, Model 642E; Port Matilda, PA, USA). The sera for analysis of IL-6 and E-selectin were stored in a freezer at a temperature of −30 °C until the moment of testing. The concentration of IL-6 was measured using the enzyme-linked immunosorbent assay (ELISA) method, with the use of a kit by R&D Systems (Abingdon, UK), catalogue (cat.) no.HS600B; the sensitivity of the method was 0.039 pg/mL, and the intra- and interassay variability were, respectively, 7.5% and 7%. The concentration range was 0.45–9.96 pg/mL. The concentration of E-selectin was measured using the ELISA method with the use of a kit from R&D Systems, cat. no.DSLE00; the sensitivity of the method was 0.009 ng/mL; and the intra- and interassay variability, respectively, were 6% and 8%. The concentration range was 17.9–79.2 ng/mL. The concentration of CRP was measured using the dry chemistry method with the use of the VITROS 5600 device (Rochester, NY, USA), the concentration of fibrinogen was measured using the optical method (device: Sysmex Ca 1500; Erlangen, Germany), the concentration of HbA1c was measured using the immunoturbidimetric method (device: VITROS 5600), the concentration of glucose was measured using the colorimetric method (device: VITROS 5600), the concentrations of total cholesterol, triglycerides, HDL-C, LDL-C, uric acid, and creatinine were measured using the dry chemistry method (device: VITROS 5600).

The weight and height of each participant were measured using a regular scale, and the body mass index (BMI) was calculated as the ratio between weight (kg) and squared height (m^2^). The glomerular filtration rate (eGFR) was calculated using the Cockcroft-Gault formula in mL/h/1.73 m^2^ (a calculation used to estimate creatinine clearance based on age, weight, serum creatinine, and gender) [18]. The concentration of HbA1c was measured in patients suffering from PAOD.

The systolic and diastolic blood pressure was measured in a supine position after at least 15 min of rest. The ABI determination was performed in the same conditions using a non-invasive method, consisting of a Doppler probe with a 10 MHz head (HADECO SMARTDOP 45, Hadeco, Inc., Miyamae-ku, Kawasaki, Japan). A sphygmomanometer was used to measure the systolic blood pressure in the arm (brachial artery) and ankle (posterior tibial and dorsalis pedis arteries), on the left and on the right side. The value of the ABI for each lower extremity was calculated based on the obtained measurements of the highest systolic blood pressure in the arm and ankle. To calculate the ABI ratio, the average value of the systolic blood pressure measurements in the ankle was divided by the average value of the systolic blood pressure measurements in the arm. After determining the ABI in the left and the right lower extremities, the lowest value was considered in the analysis of the results. A diagnosis of PAD was assigned if the ABI value was <0.9 [18].

The severity degree of the ischemia of the lower extremities was determined using Fontaine’s classification [19].

### 2.3. Statistical Calculations

The statistical calculations were carried out using the STATISTICA 7.1 PL (StatSoft, Cracow, Poland) application software and the Microsoft Excel (Microsoft Store, Cracow, Poland) spreadsheet application. The Kolmogorov–Smirnov nonparametric test was used for the verification of the assumption of the normal distribution of the analyzed variables. Two parametric tests were used to check the proposed research hypotheses: the Pearson product-moment correlation coefficient test to check the significance of the dependency, and the Student’s *t*-test to check the significance of differences of mean values between the investigated groups. Due to the fulfillment of the formal assumption of the normal distribution of all the analyzed variables, it was not necessary to use nonparametric tests based on rank statistics. The fulfillment of the assumption of the normal distribution proves the apt choice of the research sample.

For all the statistical tests, a *p*-value < 0.05 was considered as significant. The research results were presented in the form of mean ± standard deviation, and 95% confidence interval.

For the purpose of showing the relationships between the value of ABI as the dependent variable and independent variables (sex, age, the duration of PAD, body mass index (BMI), IL-6, E-selectin, fibrinogen, CRP, total cholesterol, LDL-C, HDL-C, triglycerides, uric acid, HbA1c, and eGFR), an analysis of variance (ANOVA) was performed. With the use of multiple logistic regression analysis, the influence of the independent variables on the dependent variable was estimated, and the determination coefficient was estimated to explain the variability of the ABI in the variance model used in the study. The statistical significance level was established at *p* < 0.05.

## 3. Results

The average age and BMI of the examined patients from group I were higher than in the examined people from group II and from the control group (Table 1; *p* < 0.01–0.001). The average values of the ABI were comparable in patients suffering from PAOD with and without diabetes, and the values of systolic and diastolic blood pressure were higher in patients with PAOD (groups I and II) than in the individuals from the control group (Table 1). The examined patients suffering from PAOD with diabetes (group I) and without diabetes (group II) were comparable in terms of the arterial blood pressure, the degree of ischemia of the lower extremities according to Fontaine’s classification [18], the frequency of the occurrence of comorbidities (hypertension, coronary heart disease, hyperlipidemia), smoking, and using antihypertensive, hypolipidemic, and antiplatelet medications (Table 1). Among 29 patients with PAOD and diabetes, 22 patients took insulin, 17 patients took biguanides, and four patients took derivatives of sulfonylureas (Table 1).

The average blood serum concentrations of total cholesterol, LDL-C, triglycerides, and uric acid were higher, and the concentration of HDL-C and the value of eGFR was lower in patients with PAOD (groups I and II) than in the individuals from the control group (Table 2).

**Figure 1:**
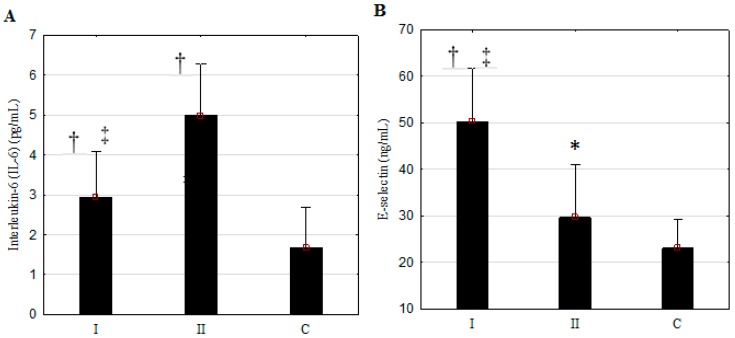
The concentrations of interleukin-6 (IL-6) (**A**) and E-selectin (**B**) in the blood serum in patients with peripheral arterial occlusive disease (PAOD) of the lower extremities; I: PAOD with diabetes, *n* = 29; II: PAOD without diabetes, *n* = 20; C: Control group, *n* = 19; † *p* < 0.001 versus Control group; * *p* < 0.05 versus Control group; ‡ *p* < 0.001 versus group II.

The examined patients suffering from PAOD with diabetes (group I) and without diabetes (group II) were comparable in terms of the values of the mentioned biochemical parameters. The concentrations of IL-6, E-selectin, and fibrinogen in the blood serum were higher in patients suffering from PAOD than in the individuals from the control group (Figure 1, Table 1; *p* < 0.05–0.001). The patients suffering from PAOD with diabetes and without diabetes differed significantly in terms of the concentration of E-selectin—which was higher—and the concentrations of IL-6 and fibrinogen—which were lower—in group I compared to group II (Figure 1, Table 2; *p* < 0.001). The concentration of CRP was only significantly elevated in patients from group I; *p* < 0.01 in reference to the control group.

The model of variance analysis used in the study included ABI as the dependent variable and sex, age, duration of PAD, BMI, IL-6, E-selectin, fibrinogen, CRP, total cholesterol, LDL-C, HDL-C, triglycerides, uric acid, HbA1c, and eGFR as the independent variables. In patients from group I, lower ABI was associated with older age ((β) = −0.65, *p* < 0.001), duration of symptoms of PAD (β = −0.33, *p* < 0.05), BMI (β = −0.82, *p* < 0.001), LDL-C (β = −0.63, *p* < 0.01), and fibrinogen (β = −0.33, *p* < 0.05); and higher ABI was associated with female sex (β = 0.60, *p* < 0.001). The calculated determination coefficients (R^2^) indicate that this model explains over 69.9% of the variance of the variable ABI (R^2^ = 0.699; F = 5.51; *p* < 0.001). In patients from group II, lower ABI was associated with greater duration of the symptoms of PAD (β = −1.31, *p* < 0.01), age (β = −0.85, *p* < 0.05), levels of CRP (β = −1.21, *p* < 0.01) and E-selectin (β = −0.43, *p* < 0.05); and greater ABI was associated with higher HDL-C (β = 1.23, *p* < 0.05) and eGFR (β = 0.69, *p* < 0.05) levels. The calculated determination coefficients (R^2^) indicate that this model explains over 61.4% of the variance in ABI (R^2^ = 0.844; F = 3.15; *p* < 0.05) (β: standardized regression coefficient; F: probability that the dependent variable equals a case, given some linear combination of the predictors).

In the examined patients from group I, correlations were found between the concentration of E-selectin and the concentrations of IL-6 (R^2^ = 0.57, *p* < 0.001), fibrinogen (R^2^ = 0.84, *p* < 0.001), CRP (R^2^ = 0.77, *p* < 0.001), triglycerides (R^2^ = 0.62, *p* < 0.001), and HDL-C (R^2^ = −0.8, *p* < 0.001); and between the concentration of IL-6 and the concentrations of fibrinogen (R^2^ = 0.42, *p* < 0.05), CRP (R^2^ = 0.44, *p* < 0.02), triglycerides (R^2^ = 0.59, *p* < 0.001), and HDL-C (R^2^ = −0.49, *p* < 0.01). In patients from group II, a negative correlation was found only between the concentration of E-selectin and the concentration of fibrinogen (R^2^ = −0.74, *p* < 0.001).

## 4. Discussion

A simple, sensitive, and commonly applied non-invasive tool for determining PAD is the ABI—especially in the case of low values (<0.9) [18]. It has been proven that low values of this indicator are a significant predictor of the morbidity and mortality of elderly people resulting from diseases of the cardiovascular system [20]. However, attention should be paid to medial arterial calcification, which occurs in some patients suffering from diabetes, and which results in an inadequate increase of the assessed ABI [21]. Our own study included patients for whom the value of ABI was <0.9, which is the criterion for diagnosing PAD.

The multiple logistic regression analysis used in the study proved that the lower value of the ABI in patients with PAOD with diabetes (and similarly without diabetes) was related to older age and the longer duration of the symptoms of ischemia of the lower extremities. Moreover, in patients with type 2 diabetes, lower ABI was related to higher values of BMI, the concentration of LDL-C, and of fibrinogen. In patients without diabetes, lower ABI was related to a higher concentration of CRP and E-selectin, and a lower concentration of HDL-C and eGFR. In patients with diabetes, the value of the ABI was related to sex, because this indicator was higher in women than in men. The results obtained in the multiple logistic regression analysis may suggest that although the value of the ABI determined in patients with PAOD was comparable in groups with and without diabetes, it was shaped by different factors in these two groups. The group of patients with diabetes was characterized by higher BMI, which—together with LDL-C and fibrinogen—constituted risk factors for the reduction of ABI. In the group of patients without diabetes, such a role may be assigned to CRP and E-selectin, which are indicators of endothelial function. Among elderly patients with newly-recognized PAD with and without diabetes, Daskalopoulou et al. [22] demonstrated a significant negative correlation between ABI and LDL-C in patients not on lipid-lowering drugs, and also between the ABI and creatinine, eGFR, CRP, and fibrinogen levels in all patients. Other researchers [23] described a significant negative correlation between ABI and CRP in patients aged below 60 with type 2 diabetes, and suggested that inflammation may play a role in the pathogenesis of atherosclerosis in these patients. Other authors did not show a correlation between the value of the ABI and the concentration of E-selectin and IL-6 in patients suffering from PAD [24].

The results obtained in the present study indicate that in patients suffering from symptomatic PAOD of the lower extremities, the concentration of the analyzed pro-inflammatory markers was increased, which suggests the participation of inflammation in the mechanism of this disease. It was demonstrated that the increase of the concentrations of pro-inflammatory markers and of the marker indicating endothelial dysfunction was diversified, depending on the co-occurrence of diabetes. The concentrations of E-selectin and CRP in the blood serum were higher, and the concentrations of IL-6 and fibrinogen were lower in patients suffering from controlled type 2 diabetes than in patients without diabetes. The observed groups of patients suffering from PAOD with and without diabetes were comparable in terms of the degree of ischemia of the lower extremities determined on the basis of the ABI and of Fontaine’s classification, in terms of the existence of comorbidities (arterial hypertension, coronary heart disease, hyperlipidemia), in terms of smoking, as well as taking antihypertensive and hypolipidemic medications. It may be assumed that the differentiated variations in the concentrations of the examined markers responsible for the development of PAOD in the observed groups or resulting from that pathology were associated with the co-occurring diabetes, and maybe with taking antidiabetic medications.

The available literature provides few reports comprising comparative studies referring to the participation of pro-inflammatory markers and markers associated with endothelial damage in the development of PAD in groups of patients with type 2 diabetes and without diabetes [11,12]. Zdrojowy et al. [11] demonstrated that in patients suffering from PAD with and without diabetes, the direction of changes of the concentrations of E-selectin and IL-6 was consistent with the findings of our research. The studies performed by Danielsson et al. [12] indicate a lack of a significant differentiation in the concentrations of IL-6 and E-selectin in patients with and without diabetes when the degree of clinical development of PAD was low. However, at an advanced-stage of the disease, at the critical stage of ischemia of the lower extremities, the concentration of IL-6 was nearly twice as high, and E-selectin levels showed an upward trend in patients with diabetes compared topatients without diabetes. Another study showed that in patients suffering from type 2 diabetes without complications, the concentration of E-selectin was significantly higher than in patients without diabetes. Nevertheless, in patients suffering from type 2 diabetes with co-occurring PAD, the concentration of E-selectin proved to be higher than in patients suffering from diabetes without PAD [13]. Additionally, in diabetic patients with severe symptomatic PAD requiring angioplasty, the concentration of E-selectin was significantly higher than in comparable patients without diabetes [14]. It is suggested that in patients suffering from diabetes, E-selectin stimulates the activity of the endothelium and becomes involved in the process of atherogenesis with the proliferation of smooth muscles, which is dependent on carbohydrate metabolism disorders.

In our own research, in patients suffering from PAOD, significant correlations were found in the group with diabetes between the concentration of E-selectin and IL-6 and the concentrations of fibrinogen, CRP, triglycerides, and HDL-C (negative correlation), as well as between the concentration of E-selectin andIL-6. In the group of patients without diabetes, a negative correlation was shown only between the concentration of E-selectin and the concentration of fibrinogen. This may result from various pathomechanisms of the development of PAOD, depending on the occurrence of diabetes. In patients with diabetes, the connections between endothelial function and the pro-inflammatory markers, the pro-thrombotic markers, and lipids in the development of atherogenic changes in the arteries of the lower extremities seem to be of more significance than in patients without diabetes. Perhaps this is associated with the damage and the dysfunction of the endothelium, which was demonstrated to be a consequence of postprandial hyperglycemia [15]. The development of PAD in diabetes is strongly associated with insulin resistance, which weakens the significance of inflammation in the pathophysiology of this disease [25]. A dysfunction of the endothelium was demonstrated in patients after coronary artery bypass grafting, which was carried out with extracorporeal circulation. The degree of this dysfunction varied in patients suffering from diabetes and in patients without diabetes. In patients suffering from diabetes, IL-6 secretion was lower, and the turnover of E-selectin was more intensified than in the patients without diabetes [26]. In examinations concerning the concentrations of pro-inflammatory markers in patients suffering from type 2 diabetes, significantly higher concentrations of IL-6, CRP, and fibrinogen were demonstrated in the case of the co-occurrence of PAD than without this disease [4,10]. Moreover, in patients suffering from both diabetes and PAD, there was a correlation between the concentration of IL-6 and the concentrations of CRP and fibrinogen [10], which was consistent with the results we obtained for patients suffering from diabetes. Subjects with PAD had elevated levels of matrix metalloproteinase-9, myeloperoxidase, IL-6, intercellular adhesion molecule-1, and high-sensitivity CRP in one examination [5], and the concentration of IL-6, CRP, and fibrinogen [6] compared with those without PAD. This observation suggests the importance of inflammatory biomarkers in the pathophysiology of PAD. PAD was associated with a higher incidence of polymorphisms of genes for IL-6 and E-selectin, the genotypes of which, respectively (GG and AA), constituted independent risk factors for the development of this disease, especially in its symptomatic form, and even at the stage of critical ischemia of the lower extremities [27].

The pathophysiology of PAD is implied by an inflammation leading to vessel damage. During the period of 12 years of observations of the progressive development of peripheral arteriosclerosis, the proatherogenic activity of pro-inflammatory markers (especially IL-6) was demonstrated [8]. Fibrinogen proved to be a significant risk factor for PAD in elderly age [2]. In our own studies, in patients with PAOD, the concentration of this parameter was elevated, wherein it was significantly more elevated in patients without diabetes than in patients with diabetes. Attention should be paid to the occurrence of a significantly negative correlation between the concentration of fibrinogen and E-selectin in patients suffering from PAOD without diabetes. CRP proved to be an independent predicting factor, strongly increasing the risk of the development of symptomatic PAD [28]. The results of our studies demonstrated that the concentration of CRP was significantly higher only in patients with diabetes suffering from PAD.

The main limitations of our work may include a small number of subjects, which was associated with their appropriate selection for the comparison groups with and without diabetes, in terms of the clinical picture (the degree of ischemia of the lower extremities), the comorbidities, the value of the ABI, smoking, and the medications taken, which allowed for the controlled treatment of arterial hypertension, hyperlipidemia, and diabetes. The patients with diabetes were on average a bit older and had higher BMI than the subjects from the non-diabetes group and the control group, which could have affected at least the ABI result, because the value of this indicator in the patients in this group demonstrated a negative correlation with age and BMI. The subjects—especially those with diabetes—were not subjected to vessel radiographs aiming at revealing the possible calcification of the vessels. However, taking into consideration the fact that for all the subjects selected for the study, the value of the ABI was <0.9, it may be assumed that none of them was affected by the phenomenon of vascular wall calcification.

## 5. Conclusions

In elderly patients suffering from PAOD of the lower extremities with type 2 diabetes and without diabetes:

1. The participation of independent factors related to the value of the ABI is diversified—in patients with diabetes, the risk factors for lower ABI are age, the duration of the symptoms of PAD, BMI, LDL-C, and fibrinogen, and the protective factor is female sex. In patients without diabetes, the risk factors for lower ABI are age, the duration of the symptoms of PAD, CRP, and E-selectin, and the value of ABI is negatively correlated with age, the duration of the symptoms of PAD, the concentration of CRP, and E-selectin; the protective factors are HDL-C and eGFR.

2. In patients with diabetes, the concentration of IL-6 and fibrinogen is lower, and the concentration of E-selectin is higher than in patients without diabetes.

## Figures and Tables

**Table 1:** Clinical parameters and ankle-brachial index (ABI) in patients with peripheral arterial occlusive disease (PAOD) of the lower extremities.

Parameter		Investigated Groups		
		Group I (PAOD with Diabetes) (*n* = 29)	Group II (PAOD without Diabetes) (*n* = 20)	Control Group (*n* = 19)
Sex (M/F)		18/11	14/6	10/9
Age (years) ^a^		70.1 ± 4.3 †,‡	65.3 ± 4.6	64.9 ± 3.5
BMI (kg/m^2^) ^a^		31.0 ± 3.8 †,$	27.8 ± 5.1	25.8 ± 2.4
Ankle-brachial index ^b^		0.78 ± 0.14 (0.7–0.88) †	0.76 ± 0.16 (0.6–0.88) †	1.12 ± 0.1 (1.1–1.2)
Systolic blood pressure (mm Hg) ^b^		132.2 ± 1.9 (131.4–132.9) †	131.6 ± 2.1 (130.6–132.6) †	126.7 ± 1.9 (125.8–127.6)
Diastolic blood pressure (mm Hg) ^b^		80.3 ± 1.9 (79.5–81) †	80.8 ± 1.9 (79.9–81.6) †	76.6 ± 2.1 (75.6–77.7)
Stage of PAD ^c^ (*n*(%))	IIa	5 (17.2)	7 (35)	-
	IIb	23 (79.3)	13 (65)	-
	III	1 (3.4)	0 (0)	-
Arterial hypertension (*n*(%))		28 (96.6)	19 (95)	0 (0)
Coronary artery disease (*n*(%))		7 (24.1)	5 (25)	0 (0)
Myocardial infarction (*n*(%))		4 (13.8)	3 (15)	0 (0)
Stroke (*n*(%))		2 (6.9)	0 (0)	0 (0)
Hyperlipidemia (*n*(%))		26 (89.7)	20 (100)	0 (0)
Smoking (*n*(%))		21 (72.4)	14 (70)	10 (52.6)
Antihypertensives (*n*(%))	ACEI/ARB	18 (62.1)	14 (70)	0 (0)
	CCB	10 (34.5)	3 (15)	0 (0)
	Beta-blockers	11 (37.9)	3 (15)	0 (0)
	Diuretics	20 (69)	10 (50)	0 (0)
Drugs for diabetes (*n*(%))	Biguanides	17 (58.6)	0 (0)	0 (0)
	Sulfonylureas	4 (13.8)	0 (0)	0 (0)
	Insulin	22 (75.9)	0 (0)	0 (0)
Other drugs (*n*(%))	Statins	29 (100)	20 (100)	0 (0)
	ASA	28 (96.6)	19 (95)	0 (0)

^a^ Data expressed as mean ± standard deviation; ^b^ data expressed as mean ± standard deviation (95% confidence interval); ^c^ according to Fontaine’s classification: IIa: Intermittent claudication after more than 200 meters of pain Free walking; IIb: Intermittent claudication after less than 200 meters of walking; III: Rest pain; † *p* < 0.001 versus Control group; ‡ *p* < 0.001 versus group II; $ *p* < 0.01 versus group II. ACEI/ARB: Angiotensin converting enzyme inhibitor/angiotensin II receptor blocker; ASA: Acetylsalicylic acid; BMI: Body mass index; CCB: Calcium channel blocker; F: Female; M: Male; PAD: Peripheral arterial disease.

**Table 2:** Biochemical parameters in patients with peripheral arterial occlusive disease of the lower extremities.

Parameter	Investigated Groups		
	Group I (PAOD with Diabetes) (*n* = 29)	Group II (PAOD without Diabetes) (*n* = 20)	Control Group (*n* = 19)
Total cholesterol (mg/dL)	177 ± 33.8 (164.1–189.8) †	182.2 ± 40.8 (166.1–198.3) †	144.8 ± 11.6 (139.3–150.4)
HDL cholesterol (mg/dL)	49.4 ± 15.7 (43.4–55.4) †	53.7 ± 19.0 (44.8–62.5) †	76.2 ± 11.4 (70.7–81.7)
LDL cholesterol (mg/dL)	103.2 ± 32.9 (90.7–115.7) †	103.3 ± 29.1 (89.7–116.8) †	64 ± 11.8 (58.3–69.7)
Triglycerides (mg/dL)	162.9 ± 58.3 (140.8–185.1) †	164.6 ± 47.4 (109.7–190.1) †	90.5 ± 17.1 (82.3–98.8)
C-reactive protein (mg/dL)	9.1 ± 5.4 (7.0–11.1) §	8.5 ± 7.2 (5.2–11.9)	5.7 ± 1.7 (4.9–6.5)
Fibrinogen (mg/dL)	377.9 ± 36.7 (344–407.7) †,‡	452.6 ± 47.4 (354.6–453.7) †	172.9 ± 15.3 (166.9–178.9)
Fasting plasma glucose (mg/dL)	116.5 ± 7.9 (93.6–117.7) †,‡	92.7 ± 6.6 (82.1–97.8)	91.8 ± 6.8 (80.3–95.9)
HbA1c (%)	6.6 ± 0.6 (6.4–6.9)	5.5 ± 0.4 (5.4–5.7)	-
Uric acid (mg/dL)	6.4 ± 1.8 (5.7–7.1) †	5.9 ± 1.1 (5.3–6.4) †	3.1 ± 3.1 (2.8–3.2; 2.9–3.2)
eGFR (mL/min/1.73 m^2^)	77.1 ± 11.7 (67.9–84.4) †	84.9 ± 15.0 (73.2–96.6) *	92.5 ± 17.2 (87.2–114.6)

Data expressed as mean ± standard deviation (95% confidence interval); † *p* < 0.001 versus Control group; § *p* < 0.01 versus Control group; * *p* < 0.05 versus Control group; ‡ *p* < 0.001 versus group II. eGFR: Estimated glomerular filtration rate; HbA1c: Glycated haemoglobin; HDL: High-density lipoprotein; LDL: Low-density lipoprotein.
